# A measurement‐based X‐ray source model characterization for CT dosimetry computations

**DOI:** 10.1120/jacmp.v16i6.5231

**Published:** 2015-11-08

**Authors:** Mitchell Sommerville, Yannick Poirier, Mauro Tambasco

**Affiliations:** ^1^ Department of Physics San Diego State University San Diego CA USA; ^2^ Department of Radiotherapy Physics CancerCare Manitoba Winnipeg MB Canada; ^3^ Department of Oncology Tom Baker Cancer Centre Calgary AB Canada

**Keywords:** computed tomography, absorbed dose, Monte Carlo, source modeling

## Abstract

The purpose of this study was to show that the nominal peak tube voltage potential (kVp) and measured half‐value layer (HVL) can be used to generate energy spectra and fluence profiles for characterizing a computed tomography (CT) X‐ray source, and to validate the source model and an in‐house kV X‐ray dose computation algorithm (kVDoseCalc) for computing machine‐ and patient‐specific CT dose. Spatial variation of the X‐ray source spectra of a Philips Brilliance and a GE Optima Big Bore CT scanner were found by measuring the HVL along the direction of the internal bow‐tie filter axes. Third‐party software, Spektr, and the nominal kVp settings were used to generate the energy spectra. Beam fluence was calculated by dividing the integral product of the spectra and the in‐air NIST mass‐energy attenuation coefficients by in‐air dose measurements along the filter axis. The authors found the optimal number of photons to seed in kVDoseCalc to achieve dose convergence. The Philips Brilliance beams were modeled for 90, 120, and 140 kVp tube settings. The GE Optima beams were modeled for 80, 100, 120, and 140 kVp tube settings. Relative doses measured using a Capintec Farmer‐type ionization chamber (0.65 cc) placed in a cylindrical polymethyl methacrylate (PMMA) phantom and irradiated by the Philips Brilliance, were compared to those computed with kVDoseCalc. Relative doses in an anthropomorphic thorax phantom (E2E SBRT Phantom) irradiated by the GE Optima were measured using a (0.015 cc) PTW Freiburg ionization chamber and compared to computations from kVDoseCalc. The number of photons required to reduce the average statistical uncertainty in dose to <0.3% was $2\times 10^{5}$. The average percent difference between calculation and measurement over all 12 PMMA phantom positions was found to be 1.44%, 1.47%, and 1.41% for 90, 120, and 140 kVp, respectively. The maximum percent difference between calculation and measurement for all energies, measurement positions, and phantoms was less than 3.50%. Thirty‐five out of a total of 36 simulation conditions were within the experimental uncertainties associated with measurement reproducibility and chamber volume effects for the PMMA phantom. The agreement between calculation and measurement was within experimental uncertainty for 19 out of 20 simulation conditions at five points of interest in the anthropomorphic thorax phantom for the four beam energies modeled. The source model and characterization technique based on HVL measurements and nominal kVp can be used to accurately compute CT dose. This accuracy provides experimental validation of kVDoseCalc for computing CT dose.

PACS numbers: 87.57.Q‐, 87.57.uq, 87.10.Rt

## I. INTRODUCTION

Since its inception, the use of computed tomography (CT) for diagnostic imaging and simulation for radiation therapy has increased,^(1)^ and it is now responsible for the majority of radiation exposure from medical imaging applications in the United States.^2^, ^3^ Exposure to ionizing radiation is associated with both deterministic and stochastic risks, though the deterministic effects are typically not expressed due to the relatively low doses associated with kV imaging modalities. The probability of carcinogenesis from diagnostic imaging however, is typically taken to be a stochastic risk that is linearly correlated to absorbed dose without requiring a threshold value.^4^, ^5^ Hence, the radiation from a CT scan permits a small, yet nonnegligible risk for cancer induction.

Mathieu et al.^(6)^ reported that intrarun, interrun, and interscanner inconsistency could cause variations in absorbed dose as high as ∼5% for a variety of scanners, though a more typical precision was ∼1%−2%. In addition, any organ in the direct path of the X‐ray beam can receive an absorbed dose in the range of 10 to 100 mGy with values varying by a factor of six between different manufacturers, and by a factor of five for the same scanner model between departments for differing scanning protocols.^(7)^ The scanning protocols define the peak X‐ray tube potential and tube current, and are chosen based on the size and material composition of the anatomical site of a patient being imaged. Due to the large variability in absorbed X‐ray dose delivered to patients being imaged and the risk associated with CT doses,^(8)^ it is important to monitor absorbed X‐ray dose in patients. This is of particular importance in the case of children who are naturally more radiosensitive.^(9)^ The ability to accurately monitor absorbed dose in individual patients would allow clinicians to assess patient‐specific carcinogenic risk and determine whether the diagnostic benefit of possible consecutive or future CT scans outweighs their risk. Furthermore, by comparing the computed dose delivered by different imaging scan protocols, one would be better‐suited to implement new protocols that limit the absorbed dose to each patient.

The computed tomography dose index volume ${\text{CTDI}}_{\text{vol}}$ is currently used to estimate radiation exposure.^(10)^ However the ${\text{CTDI}}_{\text{vol}}$ is not patient‐specific, as the phantoms used for the measurements are of fixed size, cylindrical, and homogeneous. Hence, the ${\text{CTDI}}_{\text{vol}}$ fails to account for the size, boundary contour, and tissue heterogeneity particular to an individual patient. In some cases, the ${\text{CTDI}}_{\text{vol}}$ is not an accurate metric of organ dose and so more accurate methods are needed to account for any discrepancies.^(11)^ One such discrepancy is the increase in absorbed dose to bone compared to soft tissue (at the same depth), due to the greater effective atomic number of bone and, therefore, an increased amount of photoelectric absorption. Additionally, the phantoms used to measure ${\text{CTDI}}_{\text{vol}}$ are usually smaller than the average size of an adult torso and hence tend to underestimate the scattered radiation by up to 40%.^(10)^

Boone et al.^(12)^ have recently developed several methods for determining size‐specific dose estimates by determining the effective diameter and applying conversion factors to the ${\text{CTDI}}_{\text{vol}}$. However, such techniques do not provide patient‐specific dose to regions of interest. Knowledge of the dose to regions of interest would allow clinicians to survey hotspots which could differ significantly from an averaged organ dose. An alternative approach for accurately estimating absorbed dose is to compute it using Monte Carlo (MC) methods and information from a patient's CT scan (e.g., geometry, tissue densities). However, the computational intensity involved with MC simulation is often a limiting factor for routine clinical usage. Hence, there is a need for a rapid and accurate estimation of absorbed dose to specific tissues of interest from CT imaging.

In this study, the authors use a previously developed in‐house software (kVDoseCalc)^(13)^ to compute absorbed dose from X‐rays in the kV energy range to points of interest (POIs). kVDoseCalc computes the primary component of the X‐ray dose deterministically and uses MC techniques to compute the scatter component. However, to accurately compute absorbed kV dose from the relevant physics interactions (i.e., incoherent scattering, coherent scattering, and the photoelectric effect),^(14)^ it is necessary to accurately model the X‐ray source. This includes: the angular distribution of photons emitted from the source, the energy spectra as it varies in space, and the position of the source relative to the POI.^15^, ^16^

As there is a need to assess and manage absorbed dose deposited by CT imaging, the aim of this study is twofold:

1) to demonstrate that a previously developed X‐ray virtual point source model and characterization technique involving the measurement of HVL and nominal kVp^(17)^ can be extended to model a rotating X‐ray source for the purpose of accurately computing multidetector CT dose, and
2) to experimentally validate the application of an in‐house kV X‐ray dose assessment tool (kVDoseCalc) for CT dose computation.

The authors adapted the static X‐ray source model characterization method proposed by Poirier et al.^17^, ^18^ and extended it to the CT imaging modality. This method allows one to determine spatial variations in the beam fluence and energy spectra from in‐air dose and HVL measurements, and knowledge of the kVp setting. The application to CT however, requires that the rotation of the X‐ray tube or 3D trajectory be incorporated. Such techniques are general enough to be applied to any CT scanner. Hence, the approach allows for the characterization and modeling of a CT X‐ray source for the purpose of accurately computing CT dose from a given CT scanner.

## II. MATERIALS AND METHODS

### A. kVDoseCalc description

A complete description of kVDoseCalc is given by Kouznetsov and Tambasco.^(13)^ Briefly, kVDoseCalc computes the dose at a POI or series of POIs, by numerically solving the linear Boltzmann transport equation using deterministic and stochastic methods (i.e., MC techniques) for the primary and scatter components, respectively. Practically, kVDoseCalc evaluates the incident X‐ray flux density and interaction cross‐sections at a POI. The primary flux density is governed by the inverse square law and exponential attenuation, while the scattered dose component (comprised of a first‐collision and an n‐collision contribution) is solved, using biased MC methods to generate a population of likely scattering points, effectively forming a secondary X‐ray source. These scattering points are then used to estimate the flux density at the POI through a special solution of the linear Boltzmann transport equation. kVDoseCalc has been computationally validated by comparison with the Monte Carlo N‐Particle Transport (MCNP) and Electron Gamma Shower (EGSnrc) codes using an idealized beam consisting of a single spectral static point source yielding a beam incident upon a heterogeneous block phantom.^(13)^ kVDoseCalc has also been experimentally validated for a Varian On‐Board Imager (Varian Medical Systems, Palo Alto, CA) for radiographic^(17)^ imaging using both homogeneous and heterogeneous phantoms.^17^, ^18^

kVDoseCalc requires the atomic number density, $n_{x}$, to be known for each element, x in a given material. Two transport media were considered for this study. One was a cylindrical polymethyl methacrylate (PMMA) phantom and the other was an anthropomorphic E2E SBRT phantom (a CIRS and IMT Joint Development Project). $n_{x}$ was calculated for the three atomic constituents in $\text{PMMA}C_{5}O_{2}H_{8}$ using Eq. (1):

(1) $$n_{x}=\frac{\rho \times A_{v}\times number\;of\;x\;atoms\;per\;molecule\;of\;the\;material}{A_{x}}$$

where *ρ* is the density of the material (in $g/{cm}^{3}$), $A_{v}$ is Avogadro's number, and $A_{x}$ is the atomic weight for element *x* (in g/mol). Using this equation, the atomic densities were calculated to be $5.68\times 10^{-26}$, $3.55\times 10^{-26}$, and $1.42\times 10^{-26}$ number of $\text{atoms}/{\text{cm}}^{3}$ for hydrogen, carbon, and oxygen, respectively. $n_{x}$ was similarly calculated for adult cortical (compact) bone, cancellous (spongy) bone, lung, soft tissue, and air for the anthropomorphic phantom; though instead, replacing the number of x atoms per molecule by their fractional weight. Material compositions (expressed in mass percentage) were obtained from the International Commission on Radiation Units and Measurements (ICRU) Report 44.Physical densities in $g/{\text{cm}}^{3}$ were measured using a scan of the phantom and Eclipse treatment planning software (Varian Medical Systems). kVDoseCalc is further able to map CT number ranges (in Hounsfield units) gathered from a CT image (imported in the DICOM format), to physical density ranges of a material defined by the user. kVDoseCalc uses this information to incorporate the interaction cross sections from the National Institute for Standards and Technology (NIST) for each atomic constituent.^(19)^ Individual atomic cross sections for a given interaction type (incoherent scattering, coherent scattering, and photoelectric) are then added together to determine the total cross section for the material. The total interaction cross sections, then, are utilized to calculate the dose at a POI, along with the fluence and energy.

Other parameters required for dose calculation include: the geometry of the transport medium, X‐ray source intensity consisting of a normalized 2D fluence array at the center of the CT bore (isocenter), and the source energy spectra as it varies across the lateral direction (x‐axis). It is assumed that the X‐ray source consists of a single virtual point source, located at the same spatial position as the effective focal spot on the X‐ray tube anode. The beam is assumed to be perfectly collimated such that the geometrical penumbra or scatter produced within the X‐ray tube head and collimator that may occur in reality, is modeled implicitly. It is also assumed that all of the energy lost by a photon in an interaction is absorbed locally, since fluorescence is negligible and any secondary electrons generated are absorbed at the site of interaction.^(13)^ Further, the CT X‐ray source has been modeled as a single effective virtual line source which forms a circular distribution surrounding the transport medium, thereby mimicking a rotating CT source.

### B. Source spectral characterization

Following the approach by Poirier et al.,^(18)^ the authors used third‐party MATLAB freeware Spektr^(20)^ (MathWorks, Natick, MA) to generate the energy spectra of a kV source by iteratively varying the inherent aluminum filtration in Spektr until the computed HVL converged to a measured HVL. Adapting an approach developed by Boone and Seibert,^(21)^ which consists of interpolating polynomials fitted to measured spectra, Spektr is able to determine the relative distribution of photons in 1 keV energy bins (dependent upon the kVp and aluminum filtration thickness) for the energy ranges relevant to kV imaging. Poirier et al.^(18)^ showed that the use of HVL and kVp as beam quality descriptors are reliable enough to create spectra that allow dose to be computed within 2% accuracy for kV energy beams.

In this study, the authors used the method developed by Poirier et al.^(17)^ to characterize and model the X‐ray source of a Philips Brilliance CT Big Bore (BB) 16‐slice scanner (Philips Medical Systems, Madison, WI) for beams produced by 90, 120, and 140 kVp nominal tube settings. A GE Optima CT580 scanner (GE Healthcare, Waukesha, WI) was also modeled for 80, 100, 120, and 140 kVp nominal tube settings. These CT scanners have a full bow‐tie filter that produces additional attenuation and beam hardening. To model these scanners, the authors measured the HVLs along the direction of the bow‐tie filter in 2.0 cm intervals, and interpolated between the intervals using a cubic spline function in MATLAB. HVL was measured to a distance of x=18 cm, as this is sufficient to allow for dose calculation within the phantom sizes used in this study. The authors assumed that the source spectrum is symmetric about the isocenter in the lateral direction and constant in the axial or Z direction. This has previously been shown to be a reasonable assumption, as the energy fluence per incident particle about the x‐axis is approximately symmetric.^(22)^

**Figure 1:**
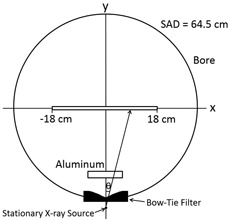
Schematic of the setup used to measure HVL along the lateral x‐axis direction for the Philips Brilliance BB scanner.

**Figure 2:**
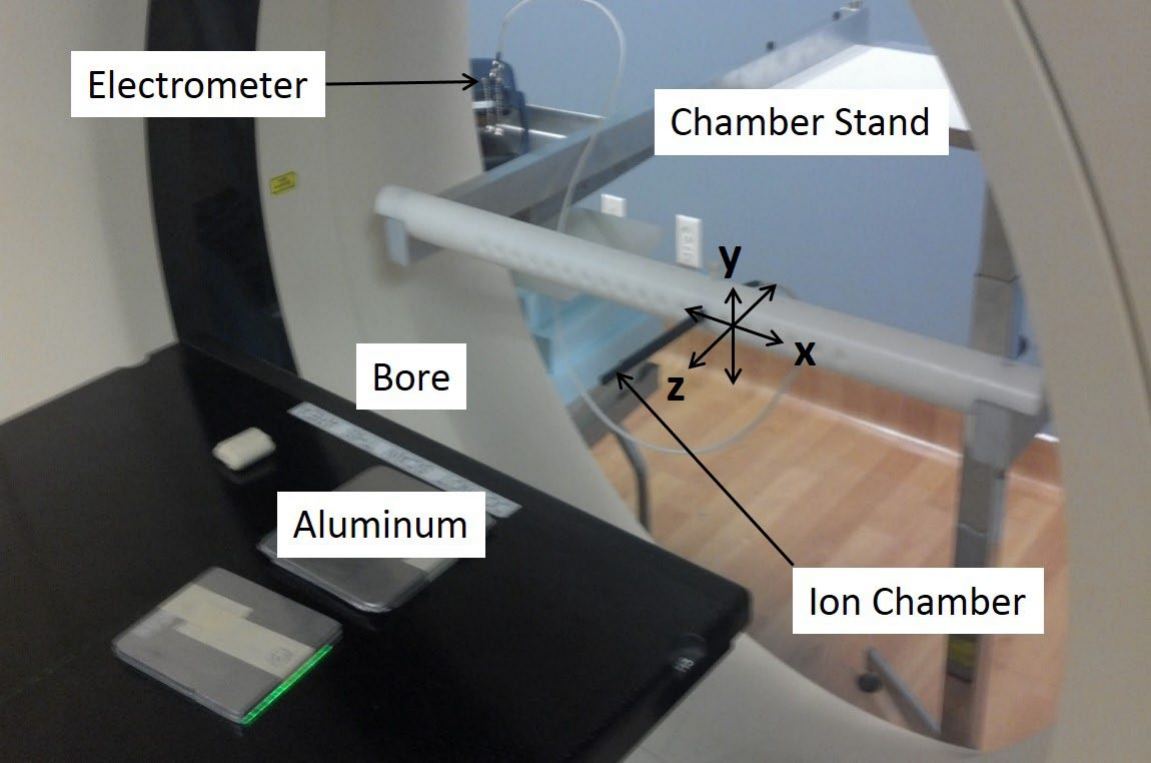
Experimental setup used to measure HVL along the lateral direction including the custom‐built chamber stand.

The HVL measurements were performed with the X‐ray tube parked at 180° (facing upwards) in pilot mode for the Philips Brilliance BB scanner. A 0.65 cc Farmer‐type PR‐06C Capintec ionization chamber (Capintec Inc., Ramsey, NJ) was attached to a stand built in‐house specifically for measuring the HVL, to ensure that the chamber would remain stationary, since the couch automatically moves in pilot mode (Fig. 1). The stand was positioned outside the beam such that the active volume of the chamber was directly inside the central axial location (z=0) of the fan beam (Fig. 2). A 75 mA tube current and a nominal beam collimation of 2.4 cm were chosen for the Brilliance BB scanner for a given energy, to maintain a reasonable signal‐to‐noise ratio (SNR).

For the GE Optima BB scanner, the chamber was attached to a stand on the couch, with the X‐ray tube parked at 90°, and with varying slabs of aluminum taped in front of the tube. The couch was raised vertically, in 2.0 cm intervals to measure the off‐axis HVLs. A 400 mA tube current and a nominal beam collimation of 2.0 cm were chosen for the Optima BB scanner for all four energies.

In‐air exposure measurements were recorded for an open beam and a beam filtered with aluminum of thickness $T_{n}$, where n=0 or 2 corresponding to the aluminum thicknesses that were slightly less and greater than the expected HVL, respectively. A two‐point nonlinear interpolation^(23)^ (Eq. (2)) was used to calculate the HVL as follows:

(2) $$HVL=\frac{T_{2,\theta }*Ln(2*\frac{D_{1}}{D_{0}})-T_{1,\theta }*Ln(1*\frac{D_{2}}{D_{0}})}{Ln(\frac{D_{1}}{D_{2}})}$$

where $T_{n,\theta }=T_{n}/cos(\theta )$ is the additional path length a photon must travel through the aluminum slabs to reach the ionization chamber located at (x, 0, 0), as shown in Figs. 1 and 2.

### C. Source fluence characterization

Beam fluence was derived by dividing the integral product of the spectra and the in‐air NIST mass‐energy attenuation coefficients^(20)^ by in‐air dose measurements along the bow‐tie filter direction (x‐axis). Since the collisional Kerma $K_{c}$ is equal to absorbed radiation dose D for the kV energies considered in this study,^(13)^ one is able to integrate the generated differential energy spectra (along with the NIST mass‐energy absorption coefficients ${\overset{ˉ}{\mu }}_{en}/\rho$ ^(20)^ and energy) to write the following relation:

(3) $$D(x,z)=K_{C}(x,z)=\int_{0}^{E_{\text{max}}}\varphi^{\prime }(x,z,E){\left[\left(\frac{{\bar{\mu }}_{en}}{\rho }\right)\right]}_{air}E\;dE$$

where *E* is the energy, ϕ′(x,z,E)=dϕ(x,z,E)/dE is the planar differential fluence, and $E_{max}$ is the maximum energy of the spectrum. It is assumed that ϕ′(x,z,E) can be written as the relative photon fluence distribution along the x‐axis, dϕ(x)/dx, multiplied by the relative photon fluence distribution along the z‐axis, dϕ(d)/dz, multiplied by the differential energy spectra, $U^{\prime }(x,z,E)$,

(4) $$\varphi^{\prime }(x,z,E)\equiv \frac{d\varphi (x)}{dx}\frac{d\varphi (z)}{dx}U^{\prime }(x,z,E)\varphi_{0}$$

where $\phi_{0}$ is a factor used to normalize to the actual ϕ′(x,z,E). Since $U^{\prime }(x,z,E)$ is the only term that depends upon photon energy, one can write the following for the x‐axis,

(5) $$\frac{D^{air}(x,z=0)}{D^{air}(x=0,z=0)}=\frac{\int_{0}^{E_{\text{max}}}\frac{d\varphi (x)}{dx}\frac{d\varphi (z=0)}{dz}U^{\prime }(x,z=0,E)\psi_{0}{\left[\left(\frac{{\bar{\mu }}_{en}}{\rho }\right)\right]}_{air}E\;dE}{\int_{0}^{E_{\text{max}}}\frac{d\varphi (x=0)}{dx}\frac{d\varphi (z=0)}{dz}U^{\prime }(x=0,z=0,E)\psi_{0}{\left[\left(\frac{{\bar{\mu }}_{en}}{\rho }\right)\right]}_{air}E\;dE}$$

Thus,

(6) $$\frac{\left(\frac{d\varphi (x)}{dx}\right)}{\left(\frac{d\varphi (x=0)}{dx}\right)}=\frac{D^{air}(x,z=0)\int_{0}^{E_{\text{max}}}U^{\prime }(x=0,z=0,E){\left[\left(\frac{{\bar{\mu }}_{en}}{\rho }\right)\right]}_{air}E\;dE}{D^{air}(x=0,z=0)\int_{0}^{E_{\text{max}}}U^{\prime }(x,z=0,E){\left[\left(\frac{{\bar{\mu }}_{en}}{\rho }\right)\right]}_{air}E\;dE}$$

Similarly, with the spectrum term being constant in the Z direction one can write,

(7) $$\frac{\left(\frac{d\varphi (z)}{dz}\right)}{\left(\frac{d\varphi (z=0)}{dz}\right)}=\frac{D^{air}(x=0,z)}{D^{air}(x=0,z=0)}$$

Therefore, one is able to derive the relative photon distribution or similarly planar fluence, along the bow‐tie filter axis from in‐air dose measurements and the energy spectra.

In this study, the authors have used the MC generated axial dose profile reported by Kim et al.^(22)^ to derive the axial fluence of a Philips Brilliance CT BB scanner using the method described above. Hence, the authors have assumed that the axial fluence field for the Brilliance CT BB scanner is not specific to the machine (or nominal kVp setting). As previously stated, the energy fluence across the axial z‐axis of the beam is approximately constant, such that the more crucial axis for determining variation of energy spectra and fluence is along the lateral bow‐tie filter axis. This is true despite the fact that the direction of the anode heel effect is along the axial z‐axis. The heel effect is negligible in this case, due to the small tilt of the anode relative to the cathode and the small beam collimations used in CT imaging. Additionally, the anode heel effect is approximately constant at any fan angle,^(24)^ resulting in an approximately constant relative axial fluence at any x‐axis position. It is worth noting that the authors used an axial fluence field for a beam with a total z‐axis profile of 2.7 cm, instead of the nominal z‐axis collimation of 2.4 cm. This was done since the authors measured the axial dose profile with radiochromic film and found that half of the dose at isocenter is absorbed approximately 1.35 cm from isocenter in the axial direction, a result which agrees with that reported by Kim et al.^(22)^ For the GE Optima scanner, the fluence was derived from the procedure detailed above and measuring the dose profile along the z‐axis at isocenter using radiochromic film. The z‐axis beam profile was extended 0.4 cm on both sides of the 2.0 cm field edges to model the penumbra effect.

The fluence along the lateral x‐axis was multiplied with the fluence along the axial z‐axis to create a normalized, discretized 2D photon fluence distribution array, which is back‐projected to the X‐ray point source location. Fluences along each axis were normalized to one, and then multiplied at each axial and lateral location. The multiplied fluence array represents the relative probability distribution of photons projected through a vacuum towards the x‐z plane at isocenter. Photons originate from the virtual point source located at the same position of the physical X‐ray source, directly above the phantom for a stationary source.

To simulate the rotation of the CT source, the trajectory and energy of a photon was randomly assigned using spectral and fluence distribution sampling functions. Following this, the photon is assigned a uniformly random starting angle using biased MC techniques. Thus, the rotation of the X‐ray source was modeled as a circular distribution of possible source coordinates, which are randomly generated along the corresponding coordinates that the physical source traverses, sampling the same fluence and spectral distribution functions at each corresponding isocenter plane perpendicular to the central axis of the source point.

### D. Sensitivity of kVDoseCalc to photons seeded

**Figure 3:**
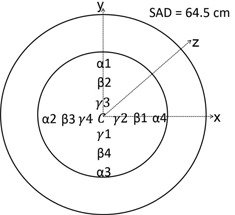
The sensitivity of kVDoseCalc to the number of photons seeded is plotted for the primary, 1st‐scattering, higher order scattering (multiscatter), and total dose components. Absolute value of the difference in dose computed relative to $2\times 10^{6}$ is shown as a percentage.

To determine the number of seeded photons necessary to reduce the statistical noise associated with dose calculation to a minimal amount, photons were seeded in progressive increments from $1\times 10^{4}$ to $2\times 10^{6}$ for simulating a circular X‐ray source incident upon the cylindrical PMMA phantom. The absolute value of the difference in dose calculated relative to the dose calculated using $2\times 10^{6}$ photons seeded is shown as a percentage of the dose calculated using $2\times 10^{6}$ seeded photons (Fig. 3). The number of photons required to reduce the average statistical uncertainty in dose to <0.3% was $2\times 10^{5}$, however some points vary by > 0.5% when less than $2\times 10^{5}$ photons were seeded. In this study we seeded $1\times 10^{6}$ photons, though similar results could have been achieved using fewer photons. For $5\times 10^{5}$, $1\times 10^{6}$, and $2\times 10^{6}$ photons seeded, the average computation time per POI was 0.88, 1.8, and 3.6 min, respectively. This demonstrates a linear relationship of computation time with number of photons seeded. Dose calculations were performed using a 4 core i7 Intel CPU (Intel Corporation, Santa Clara, CA) at 2.1 GHz with turbo boosts up to 2.9 GHz. It should also be noted that the multiscattering component of the beam contributed to the largest portion of the statistical noise (Fig. 3).

### E. Source model validation

#### E.1 PMMA phantom

**Figure 4:**
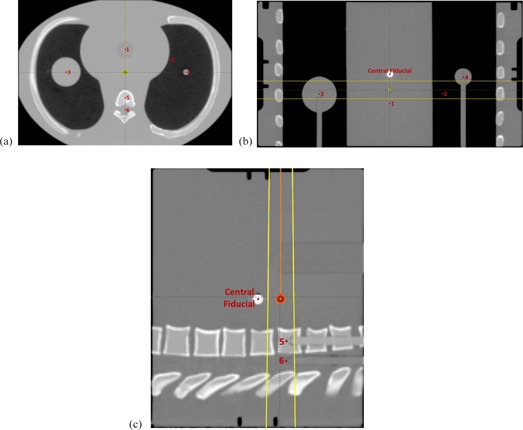
Illustration of the POIs (α, β, γ, and C) used for dose computation, which correspond to positions for inserting an ionization chamber at 1, 2.5, 4, and 7.6 cm depths in a cylindrical PMMA phantom.

Dose calculations were performed within a cylindrical homogeneous PMMA phantom with a diameter of 15.2 cm (Fig. 4). The center of the PMMA phantom was positioned at the isocenter using the laser positioning system, with a source‐to‐axis (SAD) distance of 64.5 cm for the Philips Brilliance scanner. The POIs were chosen to correspond to the ionization chamber measurement points. As the monthly quality control test tolerance of the laser system is designated to be ±1 mm, we simulated shifts in position of this amount in kVDoseCalc and found that the effect on the computed dose is negligible. This analysis also showed that the position of each voxel in which we calculate dose relative to the center corresponds to the positions we measured (with a ruler) for inserting an ionization chamber within a voxel uncertainty of ±1 (i.e., ±0.081 cm) in the x‐y plane.

We also modeled the uncertainty associated with the active volume of the ionization chamber (i.e., volume effects in nonzero dose gradient regions). The active diameter of a 0.65 cc Farmer‐type PR‐06C Capintec ionization chamber is specified as 6.4 mm;^(25)^ hence to estimate the volume effects, we calculated the standard deviation of the dose computed by kVDoseCalc at four positions (each separated by 90° angles) at the edges of the active chamber volume for each of the four radial positions that we measure dose in the phantom (Fig. 4). This was done for 90, 120, and 140 kVp beam qualities. These uncertainties were propagated and added in quadrature with the uncertainty associated with the measurement reproducibility of the ionization chamber, electrometer, and scanner to define the total measurement error bars.

To validate the beam characterization approach and the corresponding virtual X‐ray source model, the authors measured dose corresponding to positions at depths of 1, 2.5, 4, and 7.6 cm at four equally spaced positions about the center, each separated by an angle of 90° (Fig. 4). A "phantom place holder" was used to stabilize the phantom during measurement and to minimize scatter. The ionization chamber was inserted into the in‐house cylindrical PMMA phantom positioned symmetrically in the x‐y plane about the gantry isocenter. The holes for inserting the ionization chamber were drilled to the specific volume and geometry of the chamber and cylindrical pegs of the same material as the phantom were inserted into the holes that were not occupied by the chamber for a given measurement.

The electrometer was a Standard Imaging Supermax 4000 electrometer (Standard Imaging) set in the low‐dose sensitivity setting with a potential bias of +300 V. The center of the active volume of the chamber was positioned at the central axial position (z=0 cm) of the beam. We used a (300 mAs)/(400 mA) technique setting to achieve a scan length of 0.75 s in order to maintain a relatively high signal‐to‐noise ratio while minimizing the risk of overheating the X‐ray tube. A series of three dose measurements were recorded at each radial position in the phantom at 90, 120, and 140 kVp tube settings, using an axial scan consisting of four consecutive tube rotations to reduce noise. The phantom was rotated by 90° to measure all of the positions (Fig. 4). To minimize the random error associated with the phantom's positioning that might occur in such a situation, the height and position of the couch were kept constant and the overhead laser system was used to align the phantom properly along the x‐axis.

All measured doses were assumed to be the average of the three measurements and all doses were normalized to the dose at the center of the phantom. As such, the measured and computed dose comparisons were relative to a normalized center dose. To compare the dose calculations with measurement, the unbiased percent difference was calculated as follows:

(8) $$Percent\;Difference=100\%\left[\frac{\left|D_{c}-D_{m}\right|}{\left(\frac{D_{c}+D_{m}}{2}\right)}\right]$$

where *D_c_* and *D_m_* are the computed dose and measured dose relative to phantom center, respectively.

#### E.2 Anthropomorphic phantom

**Figure 5:**
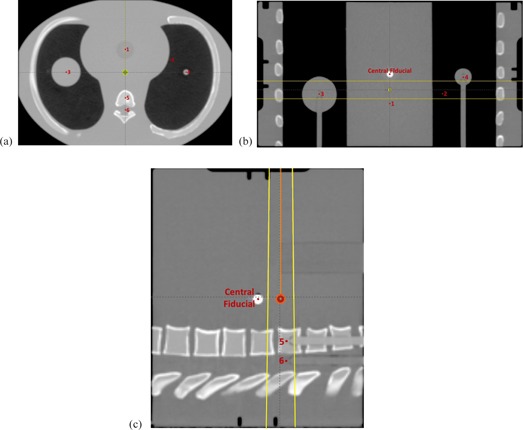
E2E SBRT Phantom shown from (a) the axial, (b) the top, and (c) the sagittal views at isocenter. The field edges (yellow) and positions of the measurement points (defined at the center of the ionization chamber) and calculation POIs are numbered 1 through 6 and correspond to soft tissue, lung, large tumor, small tumor, spinal vertebrae, and spinal cord, respectively.

Figure 5 shows the measurement and calculation POIs for the E2E SBRT Phantom. A CT scan obtained utilizing the same GE Optima BB scanner, of the phantom was acquired in axial mode at 120 kVp and 1.25 mm slice thickness, to define the transport media's geometrical and compositional properties. The E2E SBRT Phantom is comprised of articulated spine, ribs, lungs, spinal cord, vertebral body, and soft tissue. In this study, the phantom was segmented into five materials: cortical (compact) bone, cancellous (spongy) bone, lung, soft tissue, and air. As mentioned previously, HU ranges were mapped to physical density ranges and correspondingly assigned to a specific material. The materials corresponding to the six POIs in the phantom are listed in Fig. 5. The phantom was aligned using the central crosshairs and the in‐gantry lasers. A 17.5 mm couch shift towards the gantry was performed placing the active volume of the ionization chamber within the primary beam for POIs 2, 3, 5, and 6. With this positioning, POIs 1 and 4 were outside the nominal field edge, as shown in Fig. 5(b). Thus, the effective measurement point of these POIs received no primary beam dose, but they received significant patient scatter dose and dose from collimator scatter and leakage.

A PTW Freiburg (0.015 cc) pinpoint ionization chamber was connected with an IBA Dose 1 electrometer (IBA Dosimetry, Schwarzenbruck, Germany) and a voltage of +300 V was applied. A small focal spot size, 16×1.25 mm nominal beam collimation, 300 mA, and 1.0 s scanning protocol was chosen for the measurements at all four energies. The calculation voxels corresponded to the depths of the center of the active volume of the chamber upon insertion in the phantom. Since the ionization chamber was designed to measure collisional Kerma $K_{c}$ in air, conversion factors were required to determine absorbed dose in the bone‐ or lung‐equivalent materials. These factors are essentially the ratio of mass‐energy absorption coefficients of medium, m, relative to water, w, as found in Appendix B of the TG‐61 report,^(26)^ averaged over the primary photon spectrum (using HVL as the sole beam quality identifier). The mass‐energy absorption coefficient ratio of water to air was also incorporated to convert from dose in air to dose in water. The use of these factors is encumbered with some assumptions: namely, these ratios are stated for a fixed X‐ray source‐to‐POI distance (i.e., fixed energy spectrum) for a given SSD and field size, and were originally intended to be used with backscatter factors to calculate the dose at the surface of a semi‐infinite phantom of material, m. All measurements and calculations were normalized to the dose at "position 6" (Fig. 5) and relative doses were compared. This position was chosen because the active volume of the ionization chamber lies within the primary component of the beam and is centrally located within the phantom. The experimental uncertainties were calculated using standard error propagation assuming 3.0% relative dose measurement uncertainty, as given by TG‐61.

## III. RESULTS & DISCUSSION

**Figure 6:**
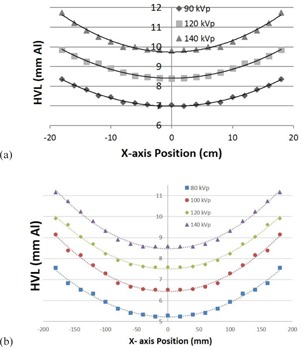
Measured HVLs as a function of lateral x‐axis position for (a) the Philips Brilliance BB scanner at 90, 120, and 140 kVp, and (b) the GE Optima BB scanner at 80, 100, 120, and 140 kVp.

**Figure 7:**
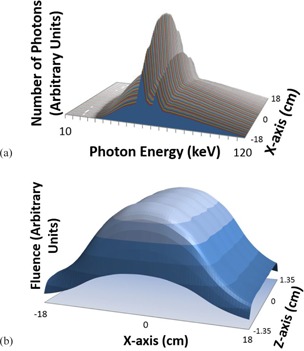
Illustration of the simplified beam model characterization including: (a) energy spectra as a function of lateral x‐axis position and (b) photon fluence in the x‐z plane at isocenter.

The HVL increased with kVp, as expected, for both the Philips Brilliance BB scanner (Fig. 6(a)) and the GE Optima BB scanner (Fig. 6(b)). The increase in the HVL along the lateral axis is a result of the beam being attenuated and hardened by the bow‐tie filter. An example of energy spectra (Fig. 7(a)) and photon fluence (Fig. 7(b)) is given for the Philips Brilliance scanner at the 120 kVp beam quality, demonstrating the typical spatial variation of these two quantities. The effects of the bow‐tie filter can be readily identified as the fluence is greatest at the central axis of the beam. The shape of the remaining beam qualities showed comparable form.

**Figure 8:**
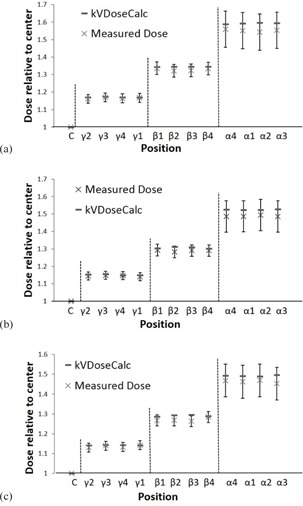
Calculated and measured relative dose for (a) 90 kVp, (b) 120 kVp, and (c) 140 kVp beam qualities at 13 positions in a cylindrical PMMA phantom for the Philips Brilliance BB scanner.

Figure 8 shows the relative doses calculated and measured for 90, 120, and 140 kVp tube settings for all the positions illustrated in Fig. 4. Though it cannot be inferred from Fig. 8, it was observed that the higher energy beams deposited more dose compared with lower energy beams. The dose from the higher energy beams is spread more uniformly throughout the phantom due to the lesser attenuation of the beam. Thus, the 90 kVp beam shows the greatest difference in dose between the R=6.2 cm α positions and the center position C (Fig. 8). It may also be noted that the error bars seen in Fig. 8 increase in value for increasing radial distance in the phantom. This is due to the fact that we are calculating dose in a region of higher dose gradient and so chamber volume effects become more important and sensitive to positioning. We observed these phenomena both computationally and with the phantom dose measurements.

The average unbiased percent difference in relative dose between computation and measurement for all 12 positions in the PMMA phantom was found to be 1.44%, 1.47%, and 1.41% for 90, 120, and 140 kVp beams, respectively. The maximum unbiased percent difference in relative dose between calculation and measurement was less than 3.50% for all energies and measurement positions. The circular symmetry of the phantom and the movement of the X‐ray source about the isocentric position of the phantom result in approximately the same absorbed dose by points at the same radial distance from the phantom center as expected.

**Figure 9:**
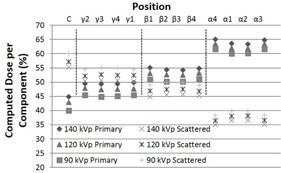
The percentage of computed primary to scattered radiation dose in the PMMA phantom by kVDoseCalc for 13 positions and three beam qualities, upon seeding the number of photons as determined in the Materials & Methods section D.

A larger percentage of the dose absorbed at the surface of the PMMA phantom is due to the primary component of the beam for all three beam qualities used in this study (Fig. 9). However, due to exponential attenuation of the primary beam, the relative contribution of the scatter component increases significantly toward the center of the phantom. It is also interesting to note that the differences in beam quality lead to a 5% dose spread in both the primary and scattered components between the 90 and 140 kVp beams, with the 140 kVp beam having relatively more primary and less scatter than the 90 kVp beam, as expected (Fig. 9).

**Figure 10:**
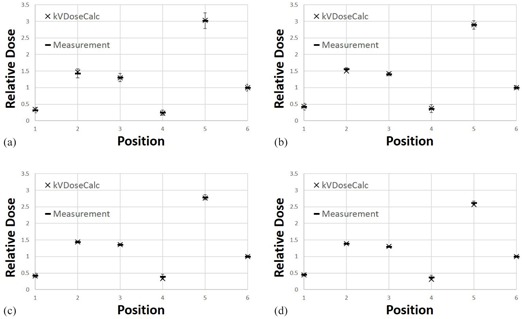
Calculated and measured relative dose for (a) 80 kVp, (b) 100 kVp, (c) 120 kVp, and (d) 140 kVp beam qualities at five positions (POIs 1 to 5) in the E2E SBRT Phantom for the GE Optima BB scanner. Note: POI 6 was used as a normalization point.

Figure 10 shows the agreement between measurement and computation of the relative dose contributions in the heterogeneous E2E SBRT Phantom. As mentioned previously, the edge of the ionization chamber volume associated with POIs 1 and 4 was partially within the beam penumbra region. Accordingly, there are chamber volume effects that were not accounted for by the dose computations because the computations were representative of absorbed dose at a single point located at the same position as the center of the ionization chamber. Hence, volume effects are expected for POIs 1 and 4 because they are in a small dose gradient region. However, these POIs are far enough from the primary field edge, making the dose gradient and associated volume effect small enough to approximate the doses by the point at the center of the ionization chamber. Furthermore, with the exception of POI 4 at 140 kVp, agreement between computation and measurement is achieved for all the doses within experimental uncertainty. The unbiased percent difference (Eq. (8)) between computation and measurement for this point that did not agree was 9.3%.

As a proof of principle, the source characterization and validation performed in this study was only carried out for a single slice (nonhelical) axial scan. However, the approach could be generalized to multislice helical scanning using knowledge of the pitch.

## IV. CONCLUSIONS

In this study, the authors have extended a previous method to characterize and model a stationary X‐ray source to that of a rotating CT source, and have shown that it can be used to accurately compute dose deposited from CT imaging. The method uses the nominal peak tube voltage potential (kVp) and spatial measurements of half‐value layer (HVL) to generate energy spectra and fluence profiles. The agreement between the experimental and computed dose results in the homogeneous cylindrical phantom and the heterogeneous anthropomorphic thorax phantom provides validation of our in‐house kV X‐ray dose calculating software (kVDoseCalc) and our beam characterization approach to model a CT source. This study represents a significant step toward developing a tool for assessing absorbed dose from a CT imaging procedure. Such a tool would aid in the management of radiation exposure from CT imaging. Future work will address the limitations of this study, including the implementation of helical scanning and automatic exposure control.

## ACKNOWLEDGMENTS

The authors would like to acknowledge Leo Moriarty and Alan Michaud for building the phantom and chamber stand used in this study and Dr. Usha Sinha who secured a travel grant (Skolil Fund – Physics Scholarship) for one of the authors (MS) to do a portion of this work at the Tom Baker Cancer Centre in Calgary, Alberta. The authors would also like to acknowledge Lindsay Schultz, Larry Burns, and Dr. Richard LaFontaine.

## Supporting information

Supplementary Material

Supplementary Material

Supplementary Material
