# Supplementary figures and images for: A measurement‐based X‐ray source model characterization for CT dosimetry computations
Source: J Appl Clin Med Phys. 2015 Nov 8;16(6):386–400. doi: 10.1120/jacmp.v16i6.5231 (PMC5691008; doi:10.1120/jacmp.v16i6.5231)

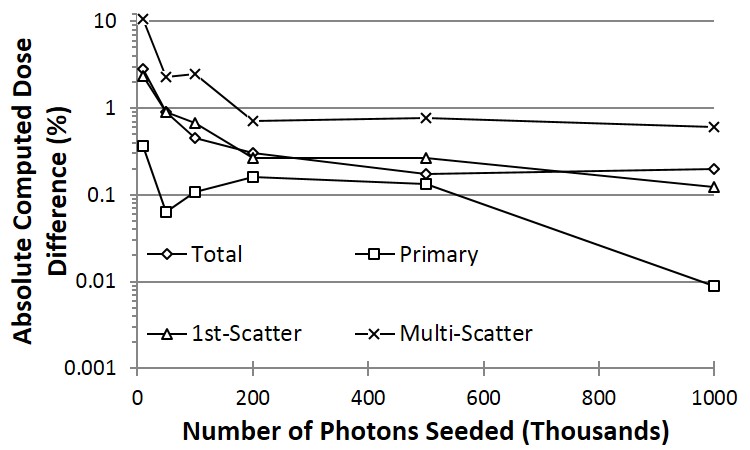

Supplement: Supplementary file 2 — Supplementary Material [file ACM2-16-386-s002.jpg]

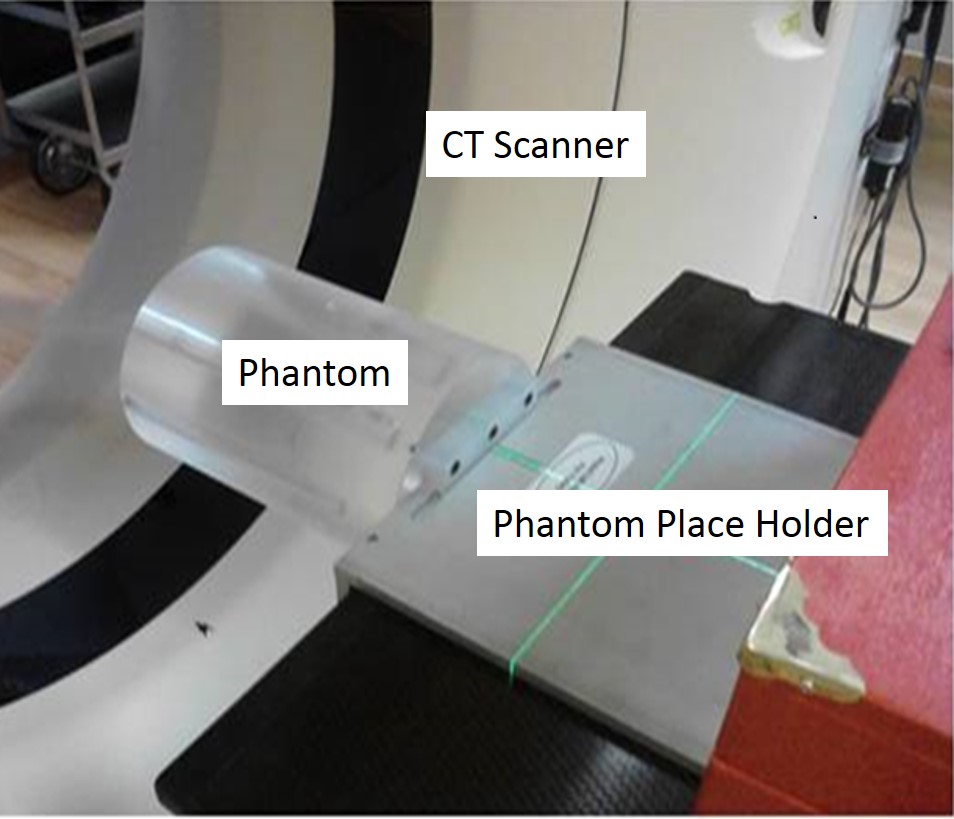

Supplement: Supplementary file 3 — Supplementary Material [file ACM2-16-386-s003.jpg]
