# Supplementary material for: A measurement‐based X‐ray source model characterization for CT dosimetry computations
Source: J Appl Clin Med Phys. 2015 Nov 8;16(6):386–400. doi: 10.1120/jacmp.v16i6.5231 (PMC5691008; doi:10.1120/jacmp.v16i6.5231)
Supplement: Supplementary file 1 — Supplementary Material [file ACM2-16-386-s001.docx]

Mauro Tambasco, Ph.D., MCCPM

San Diego State University

5500 Campanile Drive

San Diego, CA 92182-1233

July 24, 2014

Dear Editor-in-Chief:

Please consider our original research article entitled, “A measurement-based x-ray source model characterization for CT dosimetry computations,” for publication in the Journal of Applied Clinical Medical Physics. This submission describes and validates a clinically feasible approach for characterizing the spatial and spectral fluence of a CT beam and modelling the CT source for the purpose of computing patient-specific x-ray dose from CT imaging.

We believe this study represents an important contribution to the ability of performing patient-specific calculations of absorbed radiation dose from CT imaging. Thank you for considering this original article for publication in the Journal of Applied Clinical Medical Physics.

Sincerely,

Mauro Tambasco, Ph.D., MCCPM

Assistant Professor, Department of Physics, San Diego State University

Adjunct Assistant Professor, Dept. of Oncology & Physics and Astronomy, University of Calgary

E-mail: [mtambasco@mail.sdsu.edu](mailto:mtambasco@mail.sdsu.edu)

Phone: (619) 594-6161

Ps. The authors have no potential conflict of interest.
